# Supplementary material for: Molecular Basis of Multiple Mitochondrial Dysfunctions Syndrome 2 Caused by CYS59TYR BOLA3 Mutation
Source: Int J Mol Sci. 2021 May 3;22(9):4848. doi: 10.3390/ijms22094848 (PMC8125686; doi:10.3390/ijms22094848)
Supplement: Supplementary file 1 [file ijms-22-04848-s001.zip › ijms-1176504-supplementary.pdf]

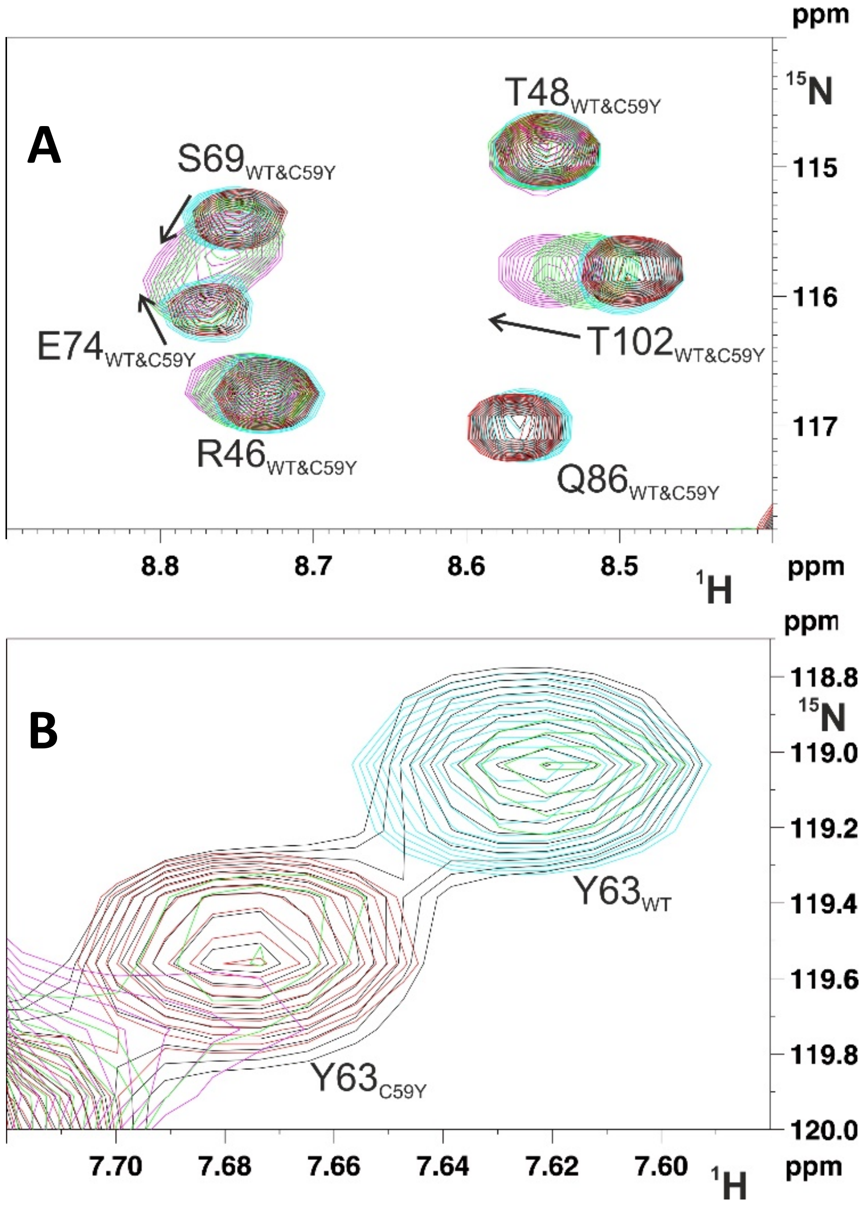

Figure S1. Estimating the relative affinity of apo GLRX5 versus BOLA3 and C59Y BOLA3 by NMR. The ^{1}H-^{15}N HSQC spectra of a 1:1 mixture of ^{15}N-labelled C59Y BOLA3 and ^{15}N-labelled BOLA3 in the presence of 0 (black), 1 (green) and 2 (purple) equivalents of unlabelled apo GLRX5. The signals of isolated ^{15}N-labelled C59Y BOLA3 and ^{15}N-labelled BOLA3 are in red and cyan, respectively. In (**A**) the backbone NH signals of the indicated residues of the two BOLA3 proteins are fully overlapped, while (B) the backbone NH signal of Y63 has a different chemical shift in the two BOLA3 proteins.

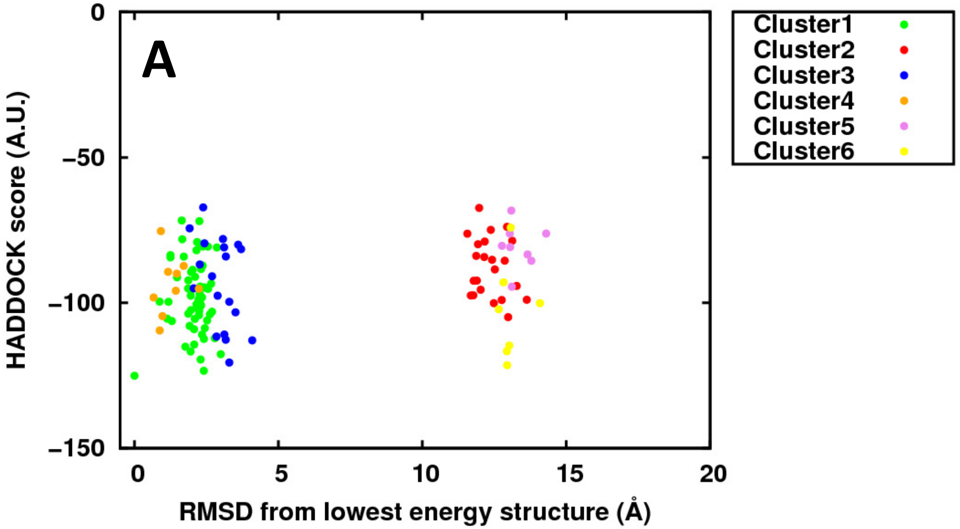

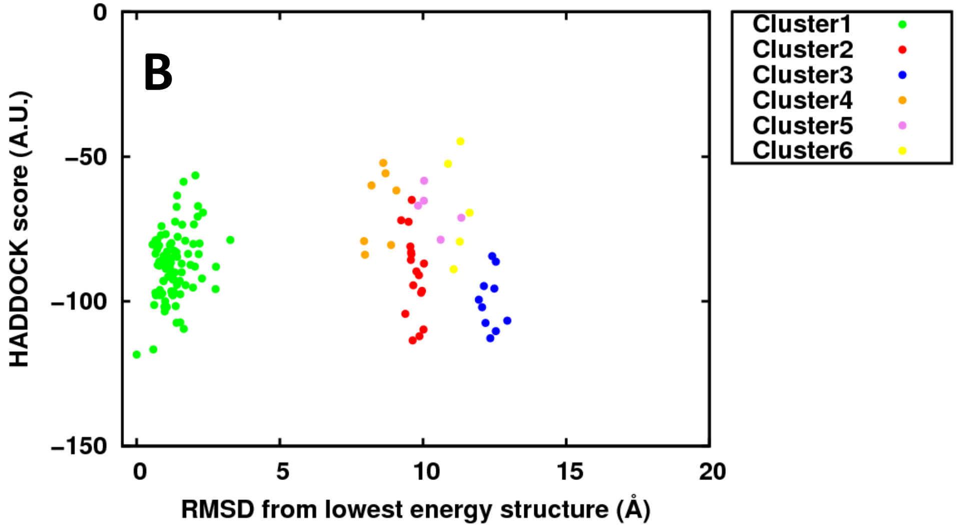

Figure S2. Clustering the HADDOCK-generated hetero-complex structural models. The plot is based on water-refined models of apo BOLA3-GLRX5 (**A**) and apo C59Y BOLA3-GLRX5 (**B**), generated by HADDOCK runs. The clusters (indicated with different colors in the plot) are plotted on the RMSD from lowest energy structure against the HADDOCK score.

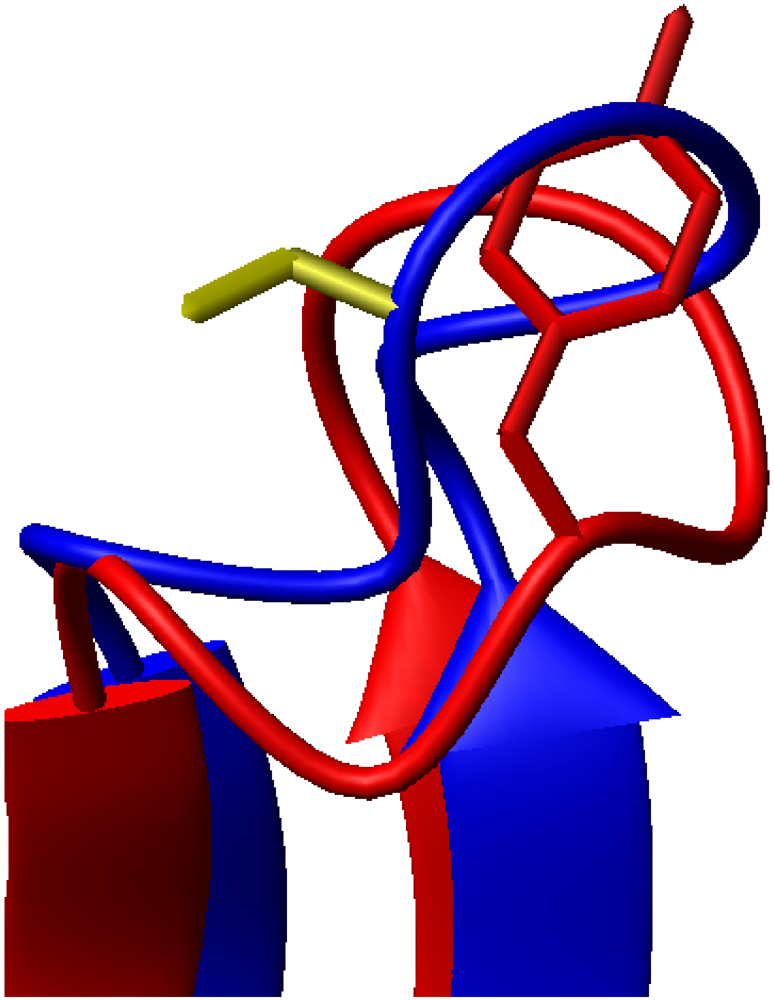

Figure S3. Structural changes of the loop containing the C59Y mutation. Ribbon diagram of the loop containing the C59Y mutation in the two structures of BOLA3 (blue) and C59Y BOLA3 (red). The side-chains of Cys 59 and Tyr 59 are shown as yellow and red sticks, respectively.

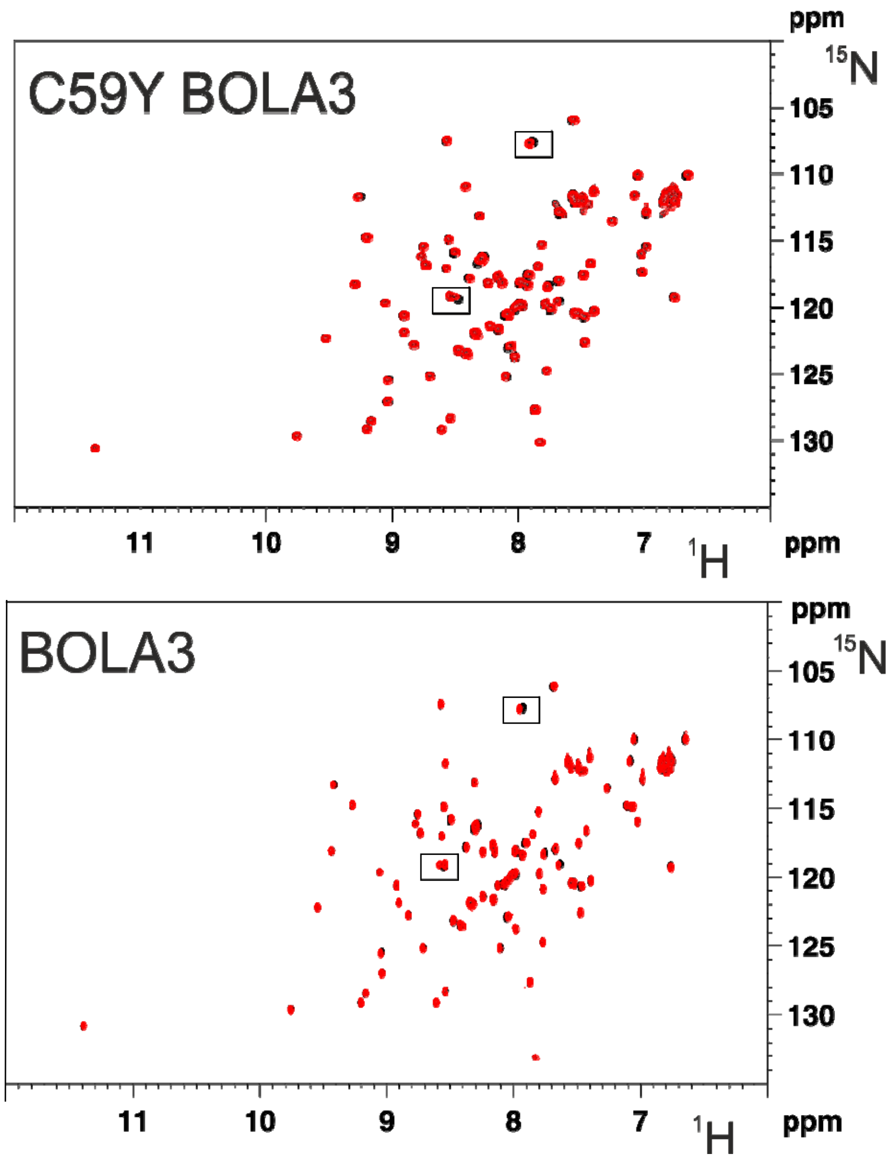

Figure S4. GSH does not interact with C59Y BOLA3 and BOLA3. ^{1}H-^{15}N HSQC NMR spectra of C59Y BOLA3 and BOLA3 with 0 mM (black) and 5 mM (red) GSH. The largest observed chemical shift differences are indicated in the boxes.

Table S1. Cluster statistics of the HADDOCK docking run for the apo BOLA3-GLRX5 complex.[a]

Cluster	HADDOCK score (a.u)	Cluster population	RMSD from the overall lowest energy structure (Å)	Van der Waals energy (Kcal mol^{-1})	Electro-static energy (Kcal mol^{-1})	Desolvation energy (Kcal mol^{-1})	Restraints violation energy (Kcal mol^{-1})	Buried Surface Area (Å^2)
1	-116.72 ±4.51	62	2.11 ±0.78	-85.52 ±6.91	-265.83 ±27.37	-11.75 ±8.21	71.37 ±36.87	1469.84 ±68.03
2	-97.22 ±3.65	22	12.43 ±0.67	-67.87 ±4.74	-232.85 ±30.8	-9.31 ±7.02	32.45 ±18.63	1252.01 ±60.84
3	-105.48 ±9.08	19	3.1 ±0.51	-69.04 ±4.37	-202.88 ±31.91	-25.68 ±7.19	95.27 ±36.04	1295.24 ±93.64
4	-93.91 ±9.45	9	1.27 ±0.46	-64.65 ±6.66	-188.58 ±64.62	-18.45 ±10.17	80.44 ±32.6	1349.18 ±72.93
5	-80.66 ±7.2	8	13.35 ±0.48	-59.48 ±3.99	-219.24 ±41.37	-3.7 ±5.62	44.37 ±26.26	1193.85 ±70.89
6	-103.17 ±15.15	7	13.08 ±0.43	-73.97 ±11.13	-133.24 ±42.63	-24.28 ±6.88	84.05 ±46.25	1435.05 ±105.65
7	-98.99 ±9.35	6	4.67 ±0.34	-62.03 ±5.3	-150.3 ±28.33	-26.31 ±4.81	43.78 ±25.23	1428.37 ±76.74
8	-79.98 ±15.74	5	2.12 ±0.41	-56.57 ±6.97	-118.88 ±25.63	-21.68 ±6.42	101.58 ±34.76	1260.05 ±101.64
9	-83.79 ±8.19	5	2.56 ±0.39	-64.52 ±8.66	-208.48 ±38.07	-8.43 ±3.58	100.08 ±20.84	1232.25 ±113.2
10	-98.27 ±11.4	5	2.53 ±0.24	-61.73 ±6.62	-161.13 ±14.66	-29.95 ±4.69	95.31 ±24.48	1395.96 ±72.04
11	-69.7 ±9.5	4	7.06 ±0.28	-48.26 ±5.31	-89.61 ±37.78	-28.3 ±2.53	158.18 ±28.71	1141.77 ±40.72
12	-76.34 ±12.4	4	14.66 ±0.11	-60.13 ±6.58	-100.78 ±33.1	-21.79 ±5.67	156.63 ±26.32	1142.25 ±100.47
13	-77.99 ±10.6	4	5.24 ±0.07	-56.76 ±8.26	-77.89 ±32.86	-26.34 ±3.81	128.93 ±22.28	1198.31 ±80.82

[a] All values are averages and standard deviation calculated from the best scoring ten models of each cluster.

Table S2. Cluster statistics of the HADDOCK docking run for the apo C59Y BOLA3-GLRX5 complex.[a]

Cluster	HADDOCK score (a.u)	Cluster population	RMSD from the overall lowest energy structure (Å)	Van der Waals energy (Kcal mol^{-1})	Electro-static energy (Kcal mol^{-1})	Desolvation energy (Kcal mol^{-1})	Restraints violation energy (Kcal mol^{-1})	Buried Surface Area (Å^2)
1	-103.56 ±6.73	60	1.75 ±0.71	-65.11 ±4.72	-296.01 ±42.13	-20.31 ±4.37	114.6 ±55.07	1010.83 ±46.1
2	-65.83 ±6.08	14	10.53 ±0.34	-40.22 ±3.71	-64.99 ±15.92	-36.01 ±6.13	168.97 ±33.77	877.72 ±57.52
3	-101.41 ±10.53	10	11.39 ±0.25	-65.92 ±9.03	-126.12 ±38.47	-32.41 ±8.26	95.33 ±33.01	1386.17 ±54.18
4	-97.32 ±15.82	9	7.38 ±0.31	-65.87 ±10.31	-150.35 ±32.03	-26.55 ±8.72	101.26 ±34.46	1482.16 ±115.73
5	-89.78 ±11.88	8	10.65 ±0.39	-58.46 ±8.78	-189.33 ±22.25	-27.02 ±7.85	146.37 ±56.88	1129.22 ±134.92
6	-62.65 ±11.14	6	8.74 ±0.45	-40.71 ±5.46	-96.36 ±26.56	-20.14 ±5.6	78.35 ±21.77	964.01 ±86.93
7	-61.16 ±11.92	6	12.04 ±0.3	-39.5 ±6.38	-84.98 ±23.88	-29.51 ±5.92	163.52 ±70.45	893.79 ±46.77
8	-81.38 ±2.49	6	6.83 ±0.16	-48.14 ±4.83	-63.54 ±15.55	-36.74 ±6.86	98.52 ±37.59	1141.8 ±41.01
9	-86.73 ±18.22	5	13.45 ±0.73	-56.49 ±8.3	-124.97 ±28.01	-33.94 ±7.74	162.03 ±55.53	1311.44 ±50.84
10	-79.07 ±8.73	4	8.72 ±0.29	-48.14 ±5	-79.42 ±17.33	-39.08 ±6	160.93 ±52	1169.44 ±46.35
11	-86.91 ±19.24	4	12.95 ±0.11	-60.26 ±5.09	-123.97 ±24.39	-25.6 ±11.89	113.38 ±52.3	1406.48 ±60.22

[a] All values are averages and standard deviation calculated from the best scoring ten models of each cluster.
